# Caloric restriction increases lifespan but affects brain integrity in grey mouse lemur primates

**DOI:** 10.1038/s42003-018-0024-8

**Published:** 2018-04-05

**Authors:** Fabien Pifferi, Jérémy Terrien, Julia Marchal, Alexandre Dal-Pan, Fathia Djelti, Isabelle Hardy, Sabine Chahory, Nathalie Cordonnier, Loïc Desquilbet, Murielle Hurion, Alexandre Zahariev, Isabelle Chery, Philippe Zizzari, Martine Perret, Jacques Epelbaum, Stéphane Blanc, Jean-Luc Picq, Marc Dhenain, Fabienne Aujard

**Affiliations:** 1 UMR CNRS/MNHN 7179, Mécanismes Adaptatifs et Evolution, 1 Avenue du Petit Château, 91800 Brunoy, France; 2 Unité d’ Ophtalmologie, Université Paris-Est, Ecole Nationale Vétérinaire d’Alfort, 7 Avenue du Général de Gaulle, 94704 Maisons-Alfort, France; 3 Histology and Pathology Department, Veterinary School of Alfort, PRES Paris Est, 94704 Maisons-Alfort, France; 4 Service de Biostatistique et d’Épidémiologie Clinique, Ecole Nationale Vétérinaire d’Alfort, Maisons-Alfort, 94704 France; 5 Université de Strasbourg, IPHC, 23 rue Becquerel, 67087 Strasbourg, France; 6 CNRS, UMR7178, 23 rue Becquerel, 67087 Strasbourg, France; 7 Unité Mixte de Recherche en Santé 894 INSERM, Centre de Psychiatrie et Neurosciences, Université Paris Descartes, Sorbonne Paris Cité, 102-108 rue de la Santé, Paris, 75014 France; 8 Laboratoire de Psychopathologie et de Neuropsychologie, EA 2027, Université Paris 8, 2 rue de la Liberté, 93000 St Denis, France; 9 Neurodegenerative Diseases Laboratory, CNRS, CEA, Université Paris-Sud, Université Paris-Saclay UMR 9199, 92265 Fontenay-aux-Roses, France; 10 Commissariat à l’Energie Atomique et aux Energies Alternatives (CEA), Direction de la Recherche Fondamentale (DRF), Molecular Imaging Research Center (MIRCen), 92265 Fontenay-aux-Roses, France

## Abstract

The health benefits of chronic caloric restriction resulting in lifespan extension are well established in many short-lived species, but the effects in humans and other primates remain controversial. Here we report the most advanced survival data and the associated follow-up to our knowledge of age-related alterations in a cohort of grey mouse lemurs (*Microcebus murinus*, lemurid primate) exposed to a chronic moderate (30%) caloric restriction. Compared to control animals, caloric restriction extended lifespan by 50% (from 6.4 to 9.6 years, median survival), reduced aging-associated diseases and preserved loss of brain white matter in several brain regions. However, caloric restriction accelerated loss of grey matter throughout much of the cerebrum. Cognitive and behavioural performances were, however, not modulated by caloric restriction. Thus chronic moderate caloric restriction can extend lifespan and enhance health of a primate, but it affects brain grey matter integrity without affecting cognitive performances.

## Abstract (web summary)

Fabien Pifferi et al. report survival and age-related brain atrophy data in grey mouse lemurs fed either a normal diet or a diet restricted in calories by 30%. They find that caloric restriction extended life span by 50% and decelerated brain white matter atrophy, but accelerated the loss of grey matter, in most of the cerebrum.

## Introduction

Caloric restriction, i.e., reducing calorie availability by ~20–50%, is one of the rare known strategies that can extend lifespan. In short-lived species such as rodents, caloric restriction can increase maximal lifespan up to 50%^1^ while improving general health and decreasing aging-associated diseases^2^. Beneficial effects of caloric restriction on age-related diseases have also been reported for long-lived species, including rhesus monkeys (*Macaca mulatta*) at the Wisconsin National Primate Research Center^3,4^ and at the National Institute on Aging^5^. Increased survival was, however, only reported in the Wisconsin National Primate Research Center study^3,4,6^. Here we examine the effects of caloric restriction on the health and lifespan of the grey mouse lemur *Microcebus murinus*, a small lemurid primate from Madagascar with a median survival in captivity of 5.7 years for males and maximum lifespan of 12 years^7^. Mouse lemurs are widely used models for human ageing. They display age-related alterations of their sensorial system, motor functions, biological rhythms and immune and endocrine systems^7^. In this species, aging leads to increased prevalence of diseases such as neoplasia or sarcopenia^8^ and glucoregulatory function alterations^9^ that also increase with aging in humans. Finally, their cerebral aging profile is similar to that of humans as they display age-related cognitive alterations associated with cerebral atrophy^10^ as well as Alzheimer’s disease-like amyloid lesions^11^. Like humans and other non-human primates, they are genetically heterogeneous providing a natural diversity of aging profiles. Because of their reduced lifespan (as compared to macaque), cohorts of calorie-restricted animals can be easily created to evaluate mechanisms leading to caloric restriction-related changes. Here we provide the first complete set of caloric restriction-related survival data for a non-human primate in association with a longitudinal follow-up of age-associated alterations in cognition and brain volumes.

In 2006, 34 captive adult male mouse lemurs (age 3.2 ± 0.1 years) were randomly assigned to either a control diet consisting of 15 g of mixture and 6 g of fresh fruit per day, equivalent to 105 kJ/day on average (control, *n* = 15) or a chronic 30% caloric restriction diet (71 kJ/day on average, *n* = 19)^12^. Their longevity, age-related pathologies, cognitive abilities, motor skills and cerebral atrophy were then followed until their natural death. Here we report that caloric restriction extended lifespan by 50% in male mouse lemurs and decelerated brain white matter atrophy but accelerated the loss of grey matter throughout most of the cerebrum.

## Results

### Caloric restriction ameliorates health and extends lifespan

**Fig. 1:**
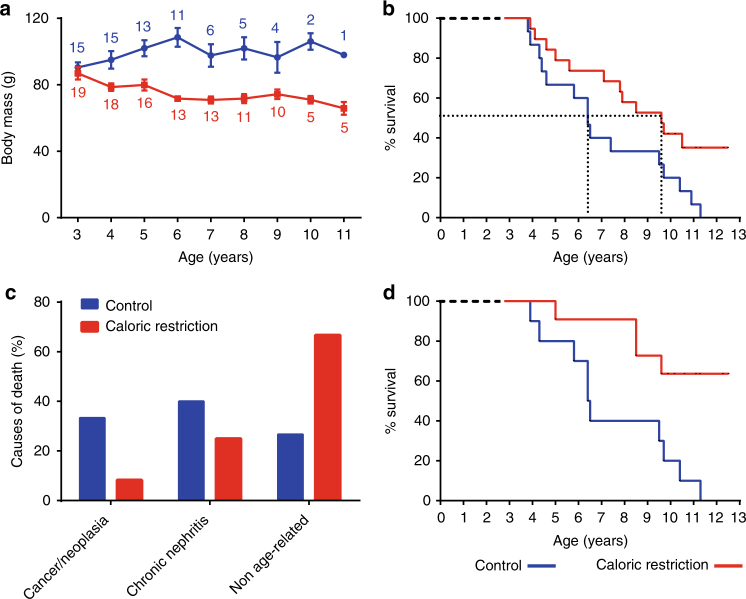
Effect of moderate caloric restriction on body mass, lifespan and age-associated pathologies in mouse lemurs. **a** Mean body mass of male mouse lemurs (*M. murinus*) at the indicated times on a chronic 30% calorie-restricted diet (*n* = 19, red) or a control diet (*n* = 15, blue). Values shown are mean ± standard error of the mean (SEM); numbers indicate the number of animals in caloric restriction and control groups. **b** Kaplan–Meier survival curves for overall mortality of the same animals shown in A (*p*-logrank = 0.02). Vertical dotted lines indicate the median survival in control (6.4 years) and calorie-restricted animals (9.6 years). **c** Incidence of the indicated age-related diseases (including cancer and nephritis) and other non-age-related causes of death (from accidents—fall with cranial trauma, MRI anesthesia incident—infections or undetermined causes) in the caloric restriction and control cohorts. The data were obtained after pathophysiological analysis of post-mortem tissues. **d** Kaplan–Meier survival curves for age-related disease mortality (*p*-logrank < 0.01)

As expected, calorie-restricted animals consistently exhibited lower body mass than the control animals, and the difference stabilised to ~28% after 4 years of treatment (70.9 ± 2.1 g versus 97.6 ± 6.8 g after 4 years of caloric restriction or control diet, respectively; linear mixed effect (LME): *F* = −6.1, *p* = 7.10e-7, Fig. 1a).

Caloric restriction had a strong positive effect on mouse lemur lifespan. At the cut-off date of the present study, none of the 15 animals on the control diet had survived, whereas 7 of the 19 (37%) calorie-restricted animals were still alive (Log-rank Mantel–Cox test: Chi-square = 4.9, *p* = 0.03; Fig. 1b). Median survival times were 6.4 and 9.6 years for control and calorie-restricted animals, respectively, which corresponds to a 50% increase in median lifespan. Also, 7 calorie-restricted animals reached 13 years of age, which is far beyond the maximal lifespan of 12 years reported in our breeding colony^7^ and of 11 years reported in the control animals. The increased lifespan of calorie-restricted individuals was accompanied by a lower incidence of age-related pathologies such as cancer and chronic nephritis (Fig. 1c) as 33.3% of deaths (4 of 12) were attributable to these diseases in the caloric restriction group compared to 73.3% of deaths (11 of the 15 deaths) in control animals (Log-rank Mantel–Cox test: Chi-square = 4.9, *p* = 0.03; Supplementary Table 1). Also, the mortality rate associated with age-related diseases was 60% lower under caloric restriction (0.44 deaths/year) compared to control animals (1.1 deaths/year; Log-rank Mantel–Cox test: Chi-square = 7.9, *p* = 0.005; Fig. 1d). However, caloric restriction did not decrease age-associated ocular diseases that involved 50 and 40% of control and calorie-restricted animals over 9 years of age, respectively, and 100% of the animals after 10 years (Supplementary Table 2).

### Caloric restriction did not alter motor and cognitive capacities

**Fig. 2:**
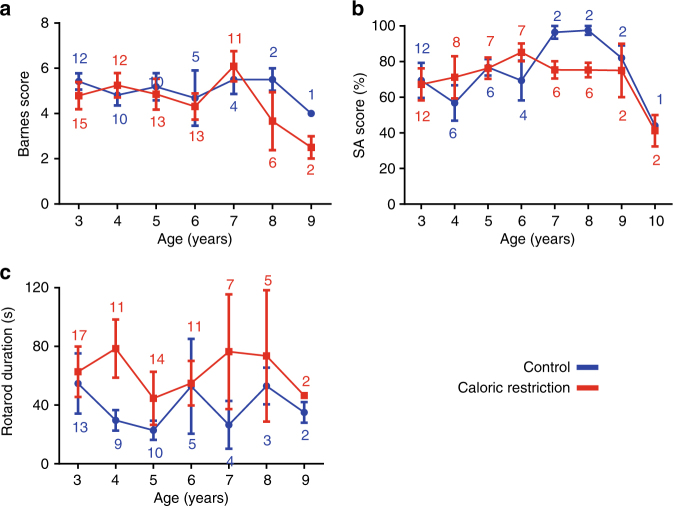
Effect of moderate caloric restriction on cognitive and motor performances in mouse lemurs. Cognitive and motor performances were tested in mouse lemurs either on a chronic 30% calorie-restricted (red) diet or on a control (blue) diet. Values shown are mean ± standard error of the mean (SEM); numbers indicate the number of animals in the calorie-restricted and control groups. **a** Barnes maze score was calculated using the following formula: Score = (10−number of errors), higher scores thus reflect better spatial memory. No significant difference in Barnes maze score was observed between control and calorie-restricted animals (*p* = 0.82). **b** The alternation score was obtained by calculating the ratio of actual alternation to possible alternation and was expressed as percentage (%), higher scores reflecting better working memory. No significant difference was observed between control and calorie-restricted animals (*p* = 0.22). **c** Motor performance during the rotarod task. No significant difference was observed between control and calorie-restricted animals (*p* = 0.54)

Although caloric restriction has been reported to protect against many aspects of brain aging in mammals^13^, deleterious effects of caloric restriction on cognitive performance have been reported for rats^14^ and for cerebral and emotional functions in humans^15,16^. Here cognitive performances were assessed by testing spatial and working memories using the Barnes maze task and the spontaneous alternation task. Age did not affect performance on the Barnes maze task (LME: *F* = 0.04, *p* = 0.57) while scores in the spontaneous alternation task were decreased over years (LME: *F* = 5.39, *p* = 0.03). However, performances in the Barnes maze task were not different between control and calorie-restricted animals (LME: *F* = 0.05, *p* = 0.82; Fig. 2a). Similarly, the spontaneous alternation task did not reveal any difference between control and calorie-restricted animals (LME: *F* = 1.63, *p* = 0.22; Fig. 2b). In several transversal studies, we reported age-related cognitive changes detected with the tasks used in the current study^10^. One possible explanation for the lack of aging effects detected here is the occurrence of practice effect that prevented detection of aging effects^17^. Motor performances, which are important for physical independence and healthy aging in humans^18^, were assessed in the accelerating rotarod task and control and calorie-restricted animals exhibited similar motor abilities (LME: *F* = 0.36, *p* = 0.54; Fig. 2c), with no effect of age in both groups (LME: *F* = 0.004, *p* = 0.95).

### Caloric restriction accelerates grey matter atrophy

**Fig. 3:**
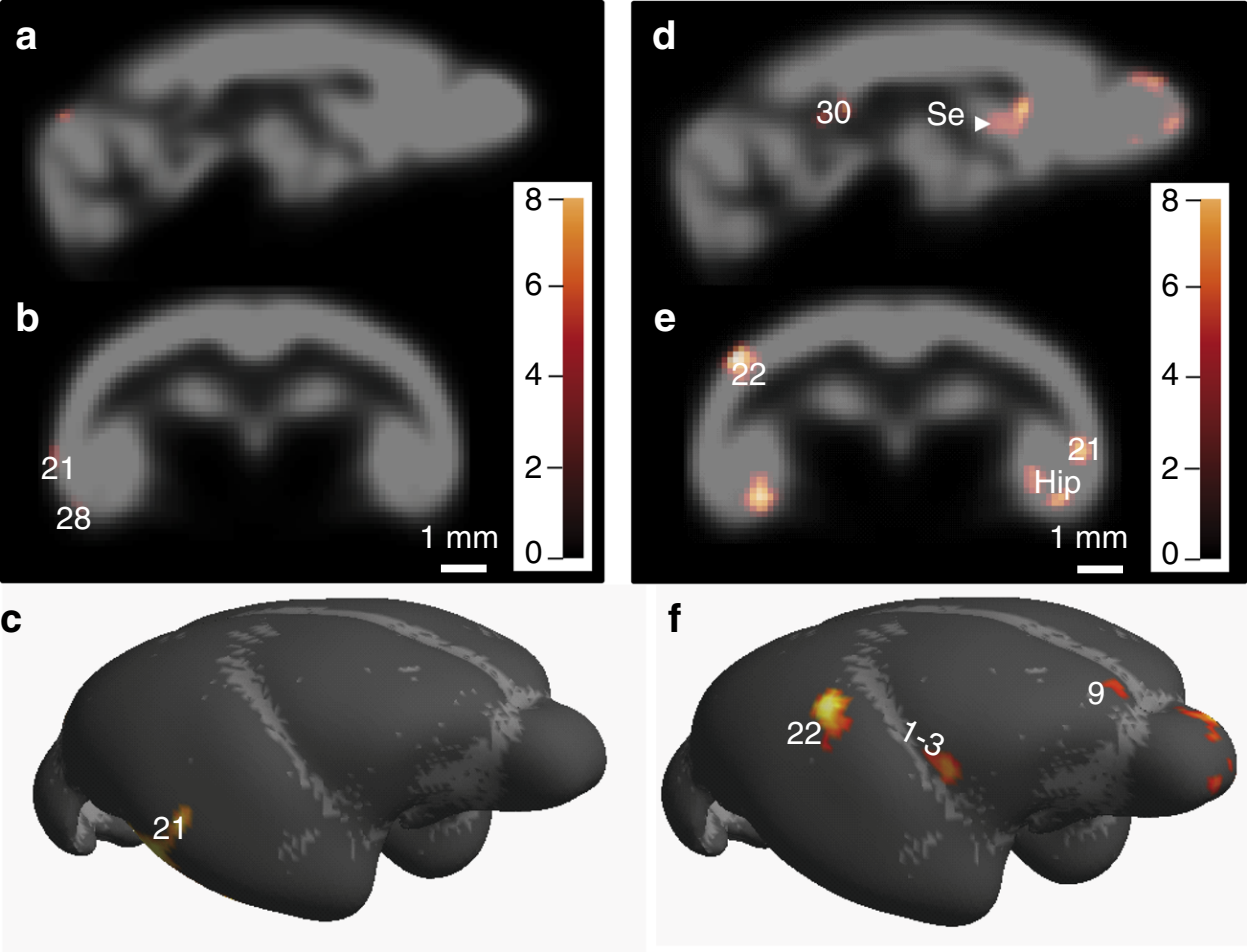
Increased grey matter atrophy in calorie-restricted compared to control mouse lemurs. Data shown at initial imaging time, i.e., after 4 years of treatment (**a**–**c**) and during the longitudinal follow-up from 7 to 10 years of age (**d**–**f**). **a** Sagittal, **b** coronal (A1.0 mm, referring to antero-posterior coordinates^23^) and **c** surface rendering representation highlighting the regions that showed stronger grey matter loss at initial imaging time in calorie-restricted compared to control animals (voxel-based morphometric analysis of serial MR images, *p* < 0.005). **d** Sagittal, **e** coronal (A1.0 mm, referring to antero-posterior coordinates^23^) and **f** surface rendering representation highlighting the regions that showed stronger age-related grey matter loss in caloric-restricted compared to control animals from 7 to 10 years of age, i.e., between 4 and 8 years after the initiation of the treatments (voxel-based morphometric analysis of time of treatment×diet group interaction, *p* < 0.005, *n* = 7 control and 13 calorie-restricted animals). The colour bars represent the value of the *t*-statistic (no unit). Numbers represent Brodmann areas of mouse lemur brain according to Brodmann and Le Gros Clark classification^24, 25^. Hip hippocampus, Se septum

**Fig. 4:**
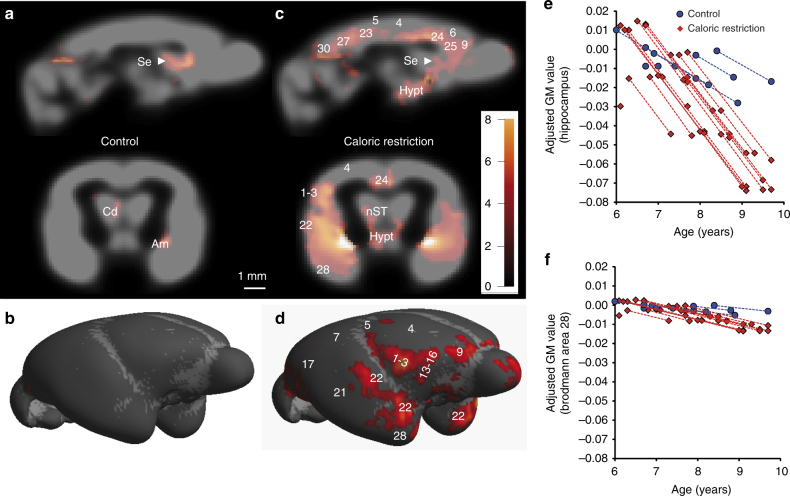
Age-associated atrophy of brain grey matter in control and caloric restricted mouse lemurs. **a**,** c** Sagittal (top) and coronal (bottom) brain representations (A3.5 mm, referring to antero-posterior coordinates^23^) views of the brains of control (**a**) and calorie-restricted (**c**) mouse lemurs showing regions with statistically significant age-related decline in grey matter volume obtained by voxel-based morphometric analysis of serial MR images (*p* < 0.005, *n* = 7 control and 13 calorie-restricted animals). Unlike control animals, calorie-restricted individuals displayed a widespread decline in grey matter throughout much of the brain. **b**,** d** Surface rendering of the data in **a**, showing regions of control (**b**) and caloric restriction brains (**d**) with age-related decline in grey matter volume. **e**, **f** Scatterplots showing changes in grey matter volume of the hippocampus (**e**) and entorhinal cortex (BA28) (**f**) during aging of control (blue, 7 contributing animals) or calorie-restricted (red, 13 contributing animals) animals. Values shown are the relative adjusted MRI grey matter values, with the values of the 6–7-year-old animals centred at 0. Dots from individual animals are connected with curves. As the data were adjusted to the general linear model after removal of confounding effects (i.e., repetition of the measures), curves from control or caloric restriction groups appear parallel. Indeed, the model estimates that the slope of the grey matter evolution is similar in control or calorie-restricted animals. It is the term $\epsilon _{j}^{k}$ corresponding to the error of the measure for each animal that is adjusted to fit the model (see Methods). The colour bar in **a**, **c** represents the value of the *t*-statistic (no unit). Numbers represent Brodmann areas (BA) of mouse lemur brain according to Brodmann and Le Gros Clark classification^24, 25^. Am medial nucleus of the amygdala, Hypt hypothalamus, nST nucleus stria terminalis, Se septum

In humans and non-human primates, including mouse lemurs, aging induces a cerebral atrophy that is associated with cognitive impairments^10,19,20^. However, this atrophy was rescued by caloric restriction in some brain regions in rhesus monkeys for both white^17^ and grey matter^3^. Serial magnetic resonance imaging (MRI) of mouse lemur brains was performed once a year for 4 years, beginning at age 7.0 ± 0.2 years. Voxel-based morphometric analysis of the initial MR images, i.e., after 4 years of treatment, revealed small regions with lower grey matter volumes in calorie-restricted animals as compared to control. The atrophied regions concerned mainly the temporal areas and entorhinal cortex (Fig. 3a–c, Supplementary Table 3). Over the subsequent 4 years, caloric restriction significantly increased the aging-associated atrophy in several other brain regions, including the hippocampus and the retrosplenial cortex (time of treatment by diet group interaction, Fig. 3d–f, Supplementary Table 4). This indicated a stronger reduction of grey matter volume with age in the caloric restriction group as compared to control animals, which is contradictory to a previous report in rhesus macaques^3,21^. Also, evaluation within each group (Fig. 4, Supplementary Tables 5 and 6) revealed that age-related grey matter atrophy occurred in widespread brain regions in calorie-restricted animals, including hippocampus (Fig. 4c–e), while only few regions, such as the septum and amygdala, were atrophied in control animals (Fig. 4a, b, Supplementary Tables 5 and 6).

**Fig. 5:**
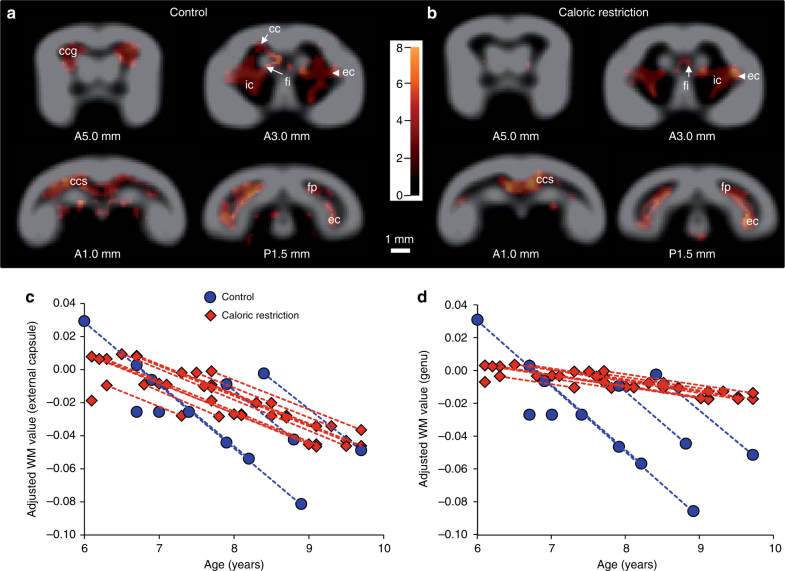
Age-associated atrophy of brain white matter in control and caloric-restricted mouse lemurs. **a**, **b** Coronal views of control (**a**) and calorie-restricted (**b**) mouse lemurs showing regions with statistically significant age-related decline in white matter volume obtained by voxel-based morphometric analysis of serial MR images (*p* < 0.005, *n* = 7 control and 13 calorie-restricted animals, brain levels refer to antero-posterior coordinates^23^). **c** Scatterplot showing changes in white matter volume of the external capsule during aging in control or calorie-restricted animals. Values shown are the relative adjusted MRI white matter values, with the values of the 6–7-year-old animals centred at 0. **d** Similar plot of white matter volumes in the genu of the corpus callosum during aging. In **c**, **d**, dots from individual animals are connected with curves. As the data were adjusted to the general linear model after removal of confounding effects (i.e., repetition of the measures), curves from the control or caloric restriction groups appear parallel. Indeed, the model estimates that the slope of the white matter evolution is similar in control or calorie-restricted animals. It is the term $\epsilon _{j}^{k}$ corresponding to the error of the measure for each animal that is adjusted to fit the model (see Methods). The colour bar represents the value of the *t*-statistic (no unit). ccg genu of the corpus callosum, cc body of the corpus callosum, ec external capsule, ic internal capsule, fi fimbria hippocampi, ccs splenium of the corpus callosum, fp posterior forceps of the corpus callosum

We did not detect any difference in white matter volume between control and calorie-restricted animals at initial imaging time. However, the slopes of age-related white matter atrophy (time of treatment by diet group interaction) over the subsequent 4 years showed a lower rate of atrophy in the genu and splenium of the corpus callosum as well as in the fimbria hippocampi of the calorie-restricted compared to control animals (Supplementary Fig. 1). These results corroborate previous studies in rhesus monkeys^21^ and mice^22^ reporting a beneficial effect of caloric restriction in preserving white matter. Evaluation of the effects of aging in each group revealed widespread loss of white matter in most parts of the corpus callosum, the internal and external capsule and the fimbria hippocampi of control animals (Fig. 5a, c, d, Supplementary Tables 7 and 8). There was similar white matter atrophy in most of these regions in the calorie-restricted animals (Fig. 5b, c, Supplementary Tables 7 and 8). However, the genu of the corpus callosum was spared in calorie-restricted animals (Fig. 5b, d, Supplementary Tables 7 and 8).

## Discussion

The present results provide evidence that chronic, moderate (30%) caloric restriction, when started early in adult life, can extend the lifespan of mouse lemurs by 50% and reduce the risk of age-associated diseases including cancer and chronic nephritis and age-associated mortality. Although this study was conducted in males only, which might moderate the translatability of these results, they support the hypothesis that caloric restriction has important beneficial effects on healthspan and lifespan in primates, as it does in many animals with a shorter lifespan. The effect of age on cognitive performances was only moderate and seemed to alter short-term working memory but not long-term spatial memory, which could be related to practice effects associated with the annual testing of the animals^17^ and to the difficulty in reliable performance of cognitive tasks in very old individuals (e.g., deterioration of motor capacities, ocular defects). Caloric restriction accelerated atrophy of grey matter in old mouse lemurs but preserved old animals from white matter atrophy compared to old controls. None of these effects of caloric restriction on brain atrophy were associated with changes in cognitive performances. Overall, this study not only sheds light on a potential negative impact of caloric restriction on brain integrity that deserves more investigation but also shows a strong positive effect of caloric restriction on enhanced physiological health ultimately leading to increased healthspan and lifespan.

## Methods

### Animals and breeding

All *M. murinus* mouse lemurs studied were males born in the laboratory breeding colony of the CNRS/MNHN in Brunoy, France (UMR 7179 CNRS/MNHN; European Institutions Agreement # E 91–114.1). Briefly, 34 male grey mouse lemurs were included in the study beginning at 3.2 ± 0.1 years of age, considered an adult age in this species in captivity. Animals were housed individually in cages (50 × 60 × 70 cm^3^) provided with wooden branches and wooden nests, in standard and constant conditions of temperature (24–26 °C), relative humidity (55%) and artificial lighting (white light, 250 lux, wavelength peak at 488 nm). Animals were fed fresh fruit and a daily mixture made up of ginger bread, cereals, milk and eggs. This diet is composed of 61% carbohydrates, 23% proteins and 16% lipids^12^. Water was given *ad libitum*. Health status of the animals was regularly checked and included weekly body weight measurement, monthly veterinarian examination and yearly ocular examination by a veterinary ophthalmologist. All described procedures were approved by the Animal Welfare board of the UMR 7179 and complied with the European ethic regulation for the use of animals in biomedical research.

### Dietary intervention

The design of the Restrikal study has been previously described^12^. At the beginning of the study, animals weighed 90 ± 5 g. They were then divided into two dietary groups that were matched for body weight, age and pedigree: a control group consisting of 15 animals fed the standard diet described above, and a group of 19 animals submitted to calorie restriction that were fed the same diet but received 30% less than the control. The daily amount of food given to the animals (15 g of mixture and 6 g of fresh fruit per day, equivalent to 105 kJ/day on average for the control group; 30% less for calorie-restricted animals) was estimated from preliminary internal studies at the Brunoy facility over a year of measuring spontaneous food intake in isolated control adult animals (unpublished data). According to the spontaneous variations of food intake in all animals^12^, the actual percentage of caloric restriction was around 24% during this study.

### Circular platform task (Barnes-like maze) for cognitive skill evaluation

The apparatus consisted of a white circular rotating platform (diameter, 100 cm) placed at 60 cm above the floor. The platform contained 12 equally spaced circular holes (each 5 cm in diameter) at 3 cm from the perimeter. A cardboard nest-box (10 × 10 × 20 cm^3^) was inserted beneath one hole and served as a refuge (goal box). A small black plywood box was placed beneath the other (non-goal) holes to prevent lemurs from jumping through these holes while permitting head entry. The platform was surrounded by a 25 cm high white wall and covered with a transparent Plexiglas cap, allowing the mouse lemurs to see cues outside the maze. The apparatus was surrounded by a black curtain hung from a square metallic frame, in the centre of which there was a one-way mirror that allowed observation. Twenty-four evenly spaced 2-Watts lights were affixed around the perimeter of the maze 50 cm above the platform to illuminate the maze. The centre of the maze was also illuminated by a 60-Watts light. Between the one-way mirror and the upper edge of the wall, various objects were attached along the inner surface of the curtain to serve as visual cues. The starting box was an open-ended dark cylinder positioned in the centre of the platform. Transparent radial Plexiglas partitions were placed between the holes to prevent the strategy used by some mouse lemurs to go directly to the periphery of the platform, then walk along the barrier wall and inspect each hole one by one. Consequently, animals had to return to the centre of the platform after each hole inspection.

Animals were given 1 day of training (day 1) and 1 day of testing (day 2). Each day comprised of four trials, each of which began with placement of the animal inside the starting box. After 30 s, the box was lifted to release the animal. For the animals, the objective was to reach the goal box positioned beneath one of the 12 holes. When it entered the goal box, the trial was stopped, and it was allowed to remain in its own nest for 1 min. After each trial, the platform was randomly rotated on its central axis to avoid the use of intra-maze cues, although the position of the goal box in the room was kept constant.

On day 1, trials 1 and 2 consisted of placing the animal in the maze centre while only one corridor, containing only the opened goal hole, was accessible (one-choice test). For trials 3 and 4, the platform comprised six reachable corridors among which only one hole was opened (six-choice test). These two trials permitted the animal to explore the maze, observe the visual cues and further learn the position of the goal box.

On day 2, all 12 corridors were accessible, with only one hole open during the four trials. Performance was assessed by the time required for the animal to reach the right exit and by the number of errors prior to reaching the goal box. An error was defined as an inspection of an incorrect hole. Only data from animals that reached the goal box before 20 min of testing were included in the behavioural analyses. This inclusion criterion and the increasing prevalence of ocular pathologies with age (Supplementary Table 2) account for the difference between the total number of animals in the study and the number of animals presented in Fig. 2a.

The parameter measured to evaluate spatial memory is the number of errors before finding the correct exit on day 2. Results are expressed as a score, calculated using the following formula: Score = (10−number of errors). A negative number gives a score of 0. Higher scores thus reflect better spatial memory.

Spatial memory was assessed from Year 1 of treatment until natural death.

### Continuous spontaneous alternation task for cognitive skill evaluation

The test was performed in a plus-maze constructed of wood (each arm: 25 cm high × 40 cm long × 15 cm wide). The four arms (labelled A, B, C and D) ended with 90° left turns (10 cm long) so that the ends of the arms were not visible from the centre of the maze to stimulate mouse lemur exploratory behaviour. In order to prevent jumps over the walls of the maze, a one-way mirror was placed on the top of the maze. This ceiling allowed experimental observation but prevented mouse lemurs from seeing extra-maze cues. Different intra-maze cues such as pieces of plastic, foam rubber or cardboard were placed on the walls of each arm in order to distinguish them. A red 15-Watts bulb was placed on the top of the longer wall of each arm and provided the only light in the room during testing. At the beginning of the trial, the animal was placed in the centre of the maze with all four arms closed by opaque doors. After 30 s, the doors were slowly raised and the mouse lemur was allowed to explore the four arms freely for 20 min. The number and the sequence of entries (all four paws into a given arm) were recorded. Alternation was defined as entry into three different arms on the same overlapping sets of four consecutive choices. For example, a set consisting of arm choices B, D, C, B, was considered as an alternation. The possible alternation sequences are equal to the number of arms entries minus three. The alternation score was obtained by calculating the ratio of actual alternation to possible alternation and was expressed as percentage (%). Only data from animals that made at least six arm entries were included in the behavioural analyses. This inclusion criterion and the increasing prevalence of ocular pathologies with age (Supplementary Table 2) account for the difference between the total number of animals in the study and the number of animals presented in Fig. 2b.

As for spatial memory, working memory was assessed from Year 1 until natural death.

### Accelerating rotarod task for motor performance evaluation

For each trial, an animal was placed on a rotarod (model 7750, Ugo Basile, Italy), a motor-driven treadmill with a 5-cm-diameter cylinder. The speed of rotation was increased from 17 to 40 rpm until the animal could no longer perform the running response without falling or gripping the rod on at least three consecutive turns, and the time spent on the cylinder was used as a measure of motor performance. Animals underwent five consecutive trials, and the best result was retained. Only data from animals that stayed on the rotarod >1 s during at least 1 trial were included in the analyses. This inclusion criterion and the increasing prevalence of ocular pathologies with age (Supplementary Table 2) account for the difference between the total number of animals in the study and the number of animals presented in Fig. 2c.

Motor performances were assessed from Year 1 of treatment until natural death.

### MRI acquisition and analysis

All the animals involved in the current study were studied by MRI from the age of 7.0 ± 0.2 years (*n* = 20 animals, 7 control, 13 calorie-restricted (7.6 ± 0.4 versus 6.8 ± 0.2 years at inclusion, respectively, Mann–Whitney *U* = 23.5, N.S.) and once a year for 4 years unless they died before. The average age of the animals at the different imaging time points was not significantly different in the two groups (8.1 ± 0.3 versus 7.8 ± 0.2 years, *U* = 218, N.S). Brain images were recorded on a 7.0 Tesla spectrometer (Varian) using a four-channel phase surface coil (RapidBiomedical, Rimpar, Germany) actively decoupled from the transmitting birdcage probe (RapidBiomedical, Rimpar, Germany). Briefly, animals were anaesthetised by isoflurane (4% for induction and 1–1.5% for maintenance). Respiratory rate was monitored to insure animal stability until the end of the experiment. Body temperature was maintained by an air-heating system. Two-dimensional fast spin-echo images were recorded with an isotropic nominal resolution of 230 µm (128 slices, TR/TE = 10000/17.4 ms; rare factor = 4; acquisition time = 32 min). MRIs were zero-filled to reach an apparent isotropic resolution of 115 µm.

Fifty-one images were analysed using voxel-based morphometry by applying SPM8 (Wellcome Trust Institute of Neurology, University College London, UK, http://www.fil.ion.ucl.ac.uk/spm/) with the SPMMouse toolbox (http://spmmouse.org) for animal brain morphometry^26^. The brain images were segmented into grey and white matter tissue probability maps using locally developed priors^26^, then spatially transformed to the standard space defined by Sawiak et al. using a grey matter mouse-lemur template^26^. Affine regularisation was set for an average-sized template, with a bias non-uniformity cut-off full-width half-maximum of 10 mm, a 5 mm basis-function cut-off and a sampling distance of 0.3 mm. The resulting grey matter and white matter portions were output in rigid template space, and DARTEL^27^ was used to create non-linearly registered maps for each subject and common templates for the cohort of animals. The warped grey matter portions for each subject were modulated with the Jacobian determinant from the DARTEL registration fields to preserve tissue amounts (‘optimised voxel-based morphometric analysis’^28^) and smoothed with a Gaussian kernel of 600 µm to produce maps for analysis.

A general linear model was evaluated with a design based on multiple regressions with the diet group effect and time of treatment of the animals of each group (control, caloric restriction) as variables of interest. This type of regression technique produces *t*-statistic and colour-coded maps that are the product of a regression model performed at every voxel in the brain. Contiguous groups of voxels that attain statistical significance, called clusters, are displayed on brain images.

With the general linear model, the brain of one animal is defined by the number “*j*”, and the location of a pixel is defined as “*k*”. The signal (i.e., the probability for the signal to be grey matter or white matter) within a pixel (${Y}_j^k$) can be explained by the following equation

$${Y}_j^k = \beta _1^k + x_{j,1}\beta _2^k + x_{j,2}\beta _3^k + T_j^1\beta _4^k + \ldots + T_j^{20}\beta _{23}^k + \mathrm{TIV}_j\beta _{24}^k + {\it{\epsilon }}_j^k$$

with ${\beta }_1^{k}$ = mean image; ${\beta }_2^{k}$ = evolution of the signal according to time of treatment for control animals (i.e., slope of signal evolution for *n* = 13 images); ${\beta }_3^{k}$ = evolution of signal according to time of treatment for calorie-restricted animals (i.e., slope of signal evolution for *n* = 38 images); $\beta _4^k$ = longitudinal follow-up for control animal #1; …; $\beta _{10}^k$ = longitudinal follow-up for control animal #7; $\beta _{11}^k$ = longitudinal follow-up for calorie-restricted animal #1; …; $\beta _{23}^k$ = longitudinal follow-up for calorie-restricted animal #13; and ${\beta }_{24}^{k}$ = effect of total intracranial volume (TIV) on the signal for each animal. In the matrix for analysis, the $T_j^x$ is 1 or 0 if the animal #*x* is analysed or not. TIV corresponds to the TIV value for each animal. It was similar for the different images from the same animal followed-up longitudinally.

The time of treatment effect within each group is defined by $x_{j,1}\beta _2^k$ and by $x_{j,2}\beta _3^k$ for the control and calorie-restricted animals, respectively. *x*_*j*,1_ and *x*_*j*,2_ represent the age of the animals in the control and caloric restriction groups, respectively. In other words, *x*_*j*,1_ = age of the animal if the *j*^th^ animal is a control animal, 0 otherwise and *x*_*j*,2_ = age of the animal if the *j*^th^ animal is a calorie-restricted animal, 0 otherwise. The term ${\it{\epsilon }}_j^k$ corresponds to the 'error' of the measure for each animal.

A contrast defines a linear combination of the *β* as *c*^*T*^*β*. For example, the time of treatment-related reduction of grey matter in the control animals would be defined using a contrast *c*^*T*^*β* = [0 −1 0…]^*T*^. The Null hypothesis is $H_0:\,c^T\beta = 0$, while the alternative hypotheses is $H_1:\,c^T\beta > 0$. This hypothesis is tested with:

$$T = \frac{{c^T\beta }}{{\sqrt {\sigma ^2c^T\left( {X^TX} \right)^{ - 1}c} }} = \frac{{{\mathrm{contrast}}}}{{\sqrt {{\mathrm{estimated}}\,{\mathrm{variance}}} }}$$

This analysis allows to remove confounding effects such as repetition of the measures during longitudinal evaluation of the same animal or TIV from the raw data ${Y}_j^k$. Voxels with a modulated grey matter value <0.2 were not considered for analysis. In other words, volumetric scans were entered as the dependent variable. Time of treatment of the animals and groups (control or caloric restriction) were the independent variables. Longitudinal follow-up effect and TIV were covariates.

One-tailed *t*-tests contrasts were set up to find areas where grey matter and white matter values were different in control and calorie-restricted animals at the beginning of the MRI study. Then other one-tailed *t*-tests were used to compare the slopes (i.e., ${\beta }_2^{k}$ for control and ${\beta }_3^{k}$ for calorie-restricted animals) of the evolution of grey matter and white matter values with aging in control and calorie-restricted animals during the 4 years of the MRI study (time of treatment×diet group interaction effect). Time of treatment effects were also evaluated in animals from the two groups. In this case, the model estimates whether the slope of the grey matter or white matter evolution within the two group (i.e., ${\beta }_2^{k}$ for control or ${\beta }_3^{k}$ = calorie-restricted animals) were different from zero. It is the term ${\it{\epsilon }}_j^k$ corresponding to the error of the measure for each animal that is adjusted to fit the model.

The threshold to consider a voxel as different between two groups was set at *p* < 0.005 (uncorrected for multiple comparisons) as in Colman et al. (2009)^3^. Clusters required 75 contiguous voxels to be selected as relevant. Clusters fulfilling these conditions were displayed on brain sections or three-dimensional views of the brain. Adjusted grey or white matter values were also presented to display time of treatment effect in control or calorie-restricted animals on which statistical analysis were performed. For each animal, they correspond to

$${Y}_j^k - \beta _1^k - {\it{\epsilon }}_j^k = x_{j,1}\beta _2^k + x_{j,2}\beta _3^k + T_j^1\beta _4^k + \ldots + T_j^{20}\beta _{23}^k + \mathrm{TIV}_j\beta _{24}^k$$

$\beta _{4\,to\,24}^k$ and TIV_*j*_ were constant for a given animal studied in a longitudinal way. Also seen in the equation, $\beta _2^k$ and $\beta _3^k$, i.e., the slopes of evolution of adjusted grey or white matter values with time were similar for the different animals from a single group (control or caloric restriction, respectively).

### Mortality data

Animals were followed until their spontaneous death. Based on specific criteria (rapid body mass loss, anaemia, difficulty breathing), euthanasia was also performed when necessary to shorten animal suffering; moribund animals were deeply anaesthetised with 100 mg/kg of pentobarbital, intraperitoneally. All organs were harvested and kept for future analysis.

### Pathophysiological analysis of post-mortem tissues

After the death of an animal, a post-mortem analysis of tissues was performed whenever possible (*n* = 13 control, *n*=11 caloric restriction). Samples from liver, kidney, spleen, small intestine, lungs, heart, stomach and pancreas were collected on each animal. Other organs (bladder, brain or colon) were collected if a macroscopic lesion was observed. All tissues were fixed in 10% neutral buffered formalin, embedded in paraffin, sectioned at 4 µm and stained with haematoxylin, eosin and saffron.

### Data analysis and statistics

Data are given as mean ± standard error of the mean (SEM). The Shapiro–Wilk goodness-of-fit test was applied to determine whether the sample data were likely to derive from a normally distributed population. Data were analysed with LME models, built with the ‘lmer’ function from package lme4 v 1.1–13 in R 3.0.2 (R Development Core Team, Vienna, Austria). Normality of models’ residuals was checked by plotting normal quantile–quantile and Q–Q line. Explanatory variables were the fixed effects of treatment (control versus caloric restriction) and of treatment duration (age effect) and their interaction. Inter-individual variability as well as repetition of measurements over years were included in the random effect. *p*-Values were calculated by performing an analysis of variance on the model using package ‘lmerTest’ v 2.0–33.

The effects of treatments (i.e., control versus caloric restriction) on both overall and age-related mortalities were investigated using Kaplan–Meier curves and Cox proportional hazard (PH) regressions. Survival time was the time between onset of treatment and any cause of death for overall mortality analyses or age-related death for age-related mortality analyses. The cut-off date was set as December 1, 2016. The PH assumption was tested by fitting a PH Cox regression with linear treatment–time interactions; these interaction terms did not significantly differ from zero for both analyses, and the proportional hazard assumptions were therefore considered as valid. SAS V9.1 (SAS Institute, Cary, NC) was used for survival analyses. Type-1 error was set at 0.05 level.

### Data availability

The data sets generated during and/or analysed during the current study are available from the corresponding author on reasonable request.

## Electronic supplementary material

Supplementary Information(PDF 169 kb)
