# Supplementary material for: Caloric restriction increases lifespan but affects brain integrity in grey mouse lemur primates
Source: Commun Biol. 2018 Apr 5;1:30. doi: 10.1038/s42003-018-0024-8 (PMC6123706; doi:10.1038/s42003-018-0024-8)
Supplement: Supplementary file 1 — Supplementary Information(PDF 169 kb) [file 42003_2018_24_MOESM1_ESM.pdf]

Supplementary Table 1. Incidence of the indicated age-related diseases (including cancer and nephritis) and other causes (from accidents, infections, or undetermined causes) in the control and calorie-restricted cohorts.

	Control		Caloric restriction	
**Cause**	**N total**	**%**	**N total**	**%**
Cancer/Neoplasia	5	**33**	1	**8**
Chronic nephritis	6	**40**	3	**25**
Non age-related	4	**27**	8	**67**

Supplementary Table 2. Prevalence of ocular pathologies (including cataracts and bilateral ocular sclerosis) observed in control and calorie-restricted animals. The numbers of animals showing ocular deficits are shown in the table as well as the total number of animals that were examined. Ocular examinations started at Year 3 of treatment.

	Control			Caloric restriction		
**Age**	**Deficits**	**N total**	**%**	**Deficits**	**N total**	**%**
6	0	9	**0**	1	14	**7**
7	0	6	**0**	0	12	**0**
8	2	4	**50**	1	9	**8**
9	1	2	**50**	2	5	**40**
10	1	1	**100**	3	3	**100**

Supplementary Table 3. Clusters of brain regions in which grey matter volumes were reduced in calorie-restricted animals as compared to controls at the first imaging time point ($p<0.005$).

**Cluster**	**T value at peak level**	**Cluster size**	**Region**
1	5.82	260	Entorhinal cortex (BA 28 - right) and middle temporal cortex (BA 21 - right)

Supplementary Table 4. Clusters of brain regions in which slopes of aging-associated grey matter loss (time of treatment x diet group interaction) were increased in calorie-restricted compared to control animals over the 4 years following the first imaging time point ($p<0.005$).

**Cluster**	**T value at peak level**	**Cluster size**	**Region**
1	6.65	264	Retrospenial cortex (left) and posterior cingulate cortex (bilateral)
2	6.10	196	Superior temporal area (BA 22 - right)
3	5.65	277	Hippocampus (left)
4	5.19	259	Hippocampus (left)
5	5.14	377	Hippocampus (right) and middle temporal area (BA 21 - right)
6	5.06	102	Septum
7	4.93	141	Precentral region (BA 4 - left)
8	4.75	88	Retrospenial cortex (right)
9	4.16	180	Postcentral region (BA 1/3 - right)
10	3.67	99	Frontal antero-ventral (BA 9 - right)

Supplementary Table 5. Clusters of brain regions showing aging-associated grey matter loss in control and calorie-restricted mouse lemurs ($p<0.005$).

**Treatment**	**Clusters**	**T value at peak level**	**Cluster size**	**Region**
Control	1	5.58	404	Septum (right)
Control	2	5.22	82	Amygdala (medial nucleus - left)
Control	3	4.76	103	Caudate (right)
Caloric restriction	1	11.83	24184	Large cluster including most brain structures (see Table S6)
Caloric restriction	2	5.53	477	Hippocampus (right)
Caloric restriction	3	4.37	268	Hippocampus (left)

Supplementary Table 6. Rate of aging-associated cerebral grey matter (GM) loss in control and calorie-restricted mouse lemurs (in percent). Measures are the slopes extracted from the scatterplot of relative adjusted GM value as a function of age and are classified from the highest to the lowest decreasing slopes for the calorie-restricted animals. *, measure performed within regions with statistically-significant age-related decline in GM volume obtained by voxel-based morphometric analysis of serial MR images ($p<0.005$).
Brodmann area (BA), from Brodmann and Le Gros Clark classification of mouse lemur brain [1,2].

**Region**	**Brodmann area**	**Rate of grey matter loss in control**	**Rate of grey matter loss in caloric restriction**
Hypothalamus	Hypothalamus	-0.55	-3.33*
Hippocampus	Hippocampus	-1.28	-2.91*
Cingulate anterior (anterior limbic area)	BA 24	-2.13	-2.06*
Posterior cingulate area (posterior limbi area)	BA 23	-0.83	-1.96*
Meynert nucl. basalis	Meynert nucl. basalis	-0.96	-1.82*
Frontal antero-ventral	BA 9	-1.11	-1.77*
Amygdala	Amygdala	-0.17	-1.61*
Cingulate anterior (pregenual area)	BA 25	-0.68	-1.57*
Frontal latero-ventral (insular area)	BA 13/16	+0.34	-1.24*
Superior temporal area	BA 22	-0.85	-1.23*
Septum	Septum	-2.46	-1.30*
Postcentral region	BA 1/3	-0.03	-1.12*
Middle temporal area	BA 21	+0.12	-0.99*
Frontal granular	BA 8	+1.71	-0.99*
Occipital area	BA 18	+1.05	-0.95
Entorhinal area	BA 28	-0.24	-0.53
Precentral region	BA 4	+0.75	-0.48
Preparietal area	BA 5	+1.62	-0.36
Parietal area	BA 7	+0.78	-0.27
Caudate	Caudate	-3.44	-0.21
Frontal dorsal agranular	BA 6	-0.58	-0.15
Striate occipital area	BA 17	-2.57	+0.92

Supplementary Table 7. Clusters of brain regions showing aging-associated white matter loss in control and calorie-restricted mouse lemurs ($p<0.005$).

**Treatment**	**Clusters**	**T value at peak level**	**Cluster size**	**Region**
Control	1	7.47	13106	Large cluster including most white matter areas (see Table S8)
Caloric restriction	1	8.09	6131	Large cluster including most white matter areas (see Table S8)
Caloric restriction	2	6.45	1411	Internal capsule

Supplementary Table 8. Rate of aging-associated cerebral white matter (WM) loss in control and calorie-restricted mouse lemurs (in percent). Measures are the slopes extracted from the scatterplot of relative adjusted WM value as a function of age and are classified from the highest to the lowest decreasing slopes for the control animals. *, measure performed within regions with statistically-significant age-related decline in WM volume obtained by voxel-based morphometric analysis of serial MR images ($p<0.005$).

**Brain region** [3]	**Rate of white matter loss in control**	**Rate of white matter loss in caloric restriction**
Corpus callosum, Perihippocampal (P0.5mm)	-9.04*	-3.66*
Corpus callosum, Splenium (A0.0mm)	-6.57*	-3.89*
Corpus callosum, Genu (A5.0 mm)	-4.26*	-0.04
Internal capsule (A3.0mm)	-3.90*	-1.33*
External capsule (A3.0mm)	-3.72*	-1.83*
Corpus callosum, Body (A4.0mm)	-2.59*	+0.02

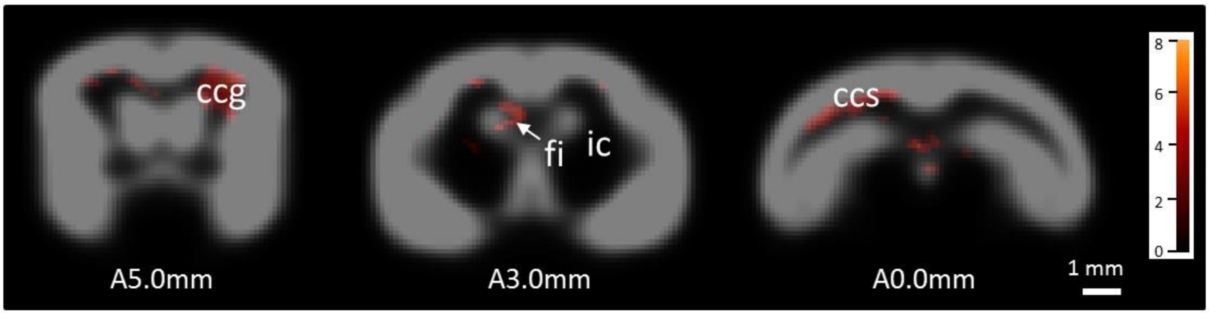

Supplementary Figure 1. Regions of age-related brain white matter preservation associated with calorie-restricted diets in mouse lemurs during the longitudinal follow-up from 4 to 8 years of treatment. The coronal views highlights regions that showed less age-related white matter loss in calorie-restricted compared to control animals after voxel-based morphometric analysis of serial MR images (time of treatment x diet group interaction, $p<0.005$, $n=7$ control and 13 calorie-restricted animals, brain levels refer to antero-posterior coordinates[3]). The color bar represents the value of the t statistic (no unit). ccg: genu of the corpus callosum; ccs: splenium of corpus callosum; fi: fimbria hippocampi. ic: internal capsule.

Supplementary references

1. Brodmann, K. (1999 (original in 1909)). Brodmann's localisation in the cerebral cortex [Vergleichende Lokalisationslehre der Grosshirnrinde in ihren Orinzipien dargestellt auf Grund des Zellenbaus]. London, Imperial College Press, Translated and edited by L.J. Garey.
2. Le Gros Clark, W. E.. The brain of *Microcebus murinus*. *Proceedings of the Zoological Society of London*. **101**, 463-485 (1931).
3. Bons, N., Silhol, S., Barbié, V., Mestre-Francés, N. & Albe-Fessard, D. A stereotaxic atlas of the grey lesser mouse lemur brain (*Microcebus murinus*). *Brain Res. Bull.* **46,** 1–173 (1998).
